# Scalable Electrophysiology in Intact Small Animals with Nanoscale Suspended Electrode Arrays

**DOI:** 10.1038/nnano.2017.55

**Published:** 2017-04-17

**Authors:** Daniel L. Gonzales, Krishna N. Badhiwala, Daniel G. Vercosa, Ben W. Avants, Zheng Liu, Weiwei Zhong, Jacob T. Robinson

**Affiliations:** 1 Applied Physics Program, Rice University, 6100 Main St., Houston, TX 77005, USA; 2 Department of Electrical and Computer Engineering, Rice University, 6100 Main St., Houston, TX 77005, USA; 3 Department of Bioengineering, Rice University, 6100 Main St., Houston, TX 77005, USA; 4 Department of BioSciences, Rice University, 6100 Main St., Houston, TX 77005, USA; 5 Department of Neuroscience, Baylor College of Medicine, One Baylor Plaza, Houston, TX 77030, USA

## Abstract

Electrical measurements from large populations of animals would help reveal fundamental properties of the nervous system and neurological diseases. Small invertebrates are ideal for these large-scale studies; however, patch-clamp electrophysiology in microscopic animals typically requires low-throughput and invasive dissections. To overcome these limitations, we present nano-SPEARs: suspended electrodes integrated into a scalable microfluidic device. Using this technology, we have made the first extracellular recordings of body-wall muscle electrophysiology inside an intact roundworm, *Caenorhabditis elegans*. We can also use nano-SPEARs to record from multiple animals in parallel and even from other species, such as *Hydra littoralis*. Furthermore, we use nano-SPEARs to establish the first electrophysiological phenotypes for *C. elegans* models for Amyotrophic Lateral Sclerosis and Parkinson’s disease, and show a partial rescue of the Parkinson’s phenotype through drug treatment. These results demonstrate that nano-SPEARs provide the core technology for microchips that enable scalable, *in vivo* studies of neurobiology and neurological diseases.

For the past century, millimeter-scale model organisms that can be rapidly raised and tested in a laboratory have helped reveal fundamental principles of genetics, development, and aging^1^. However, only recently have these advantages been combined with electrical measurements to study the nervous system^2–5^. Less than 20 years ago, scientists achieved the first single-cell electrophysiological recordings in *C. elegans* neurons by performing a manual dissection and aligning a glass micropipette electrode to the cell of interest for patch-clamp measurements^2^. Similar procedures have recorded from *C. elegans* muscle cells^5^ as well as cells in fruit flies^3^ and zebrafish larvae^4^; however, in all cases these measurements require highly invasive dissections performed by skilled researchers. These time consuming, manual dissection procedures limit scientists to only a few measurements per day with no clear path toward increased throughput^2–5^. Furthermore, because these animals are so small, any fluid expelled during dissections may significantly alter the natural concentrations of ions and signaling molecules. This change in physiology may contribute to signal degradation, such as the loss of recordings seen after just a few minutes of micropipette measurements from *C. elegans* body-wall muscles^6^.

Recently developed optical recording methods based on calcium- and voltage-sensitive fluorescence can record a proxy for electrical activity of individual cells in intact *C. elegans*^7–18^; however, compared to electrical recordings, optical measurements typically have a lower signal-to-noise ratio and reduced temporal resolution^19^. As a result, optical methods have yet to resolve individual action potentials (APs) or synaptic potentials in *C. elegans*^7–18^.

As an alternative to single-cell measurements, one can use suction pipettes or microfluidic channels to record the ensemble electrical activity from the group of cells that compose the *C. elegans* pharynx (the organ responsible for feeding)^20,21^. This approach provides a scalable method to study extracellular electrophysiology in intact *C. elegans* and screen for drugs that affect this activity^21^; however, these measurements are limited to the pharynx in nematode-like organisms. A scalable method for electrophysiology in a variety of small animals combined with the suite of high-throughput technologies to measure anatomical^22,23^ and behavioral^13,24^ properties would provide unprecedented opportunities to study fundamental neurobiology and neurological disease models.

## nano-SPEARs for small organism electrophysiology

**Figure 1:**
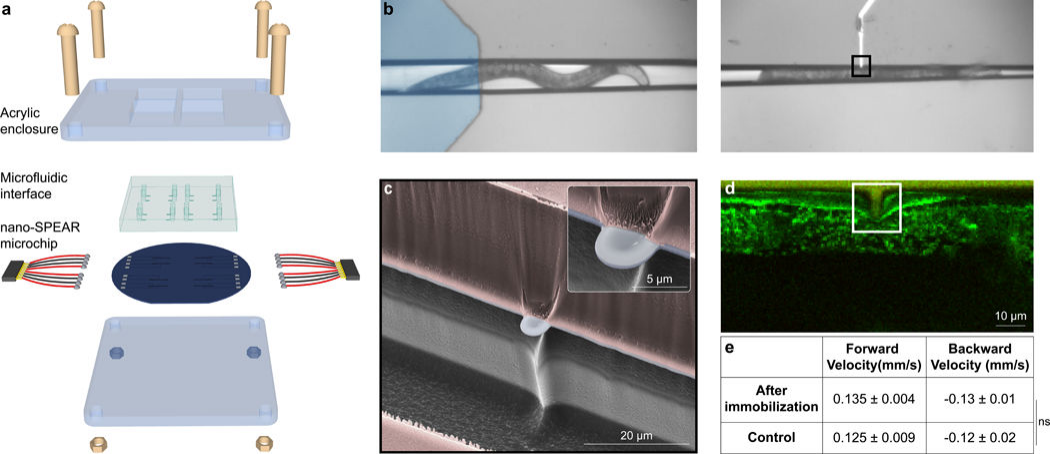
nano-SPEARs probe muscle cells in intact *C. elegans* **a,** 3D model showing all components of a nano-SPEAR electrophysiological platform. An acrylic enclosure seals a microfluidic layer to a microfabricated silicon (Si) or glass substrate. These silicon or glass substrates contain recording chambers featuring cellular-scale suspended electrodes. Wires (black and red) bonded to the nano-SPEAR chip transmit the electrophysiological signals to an amplifier and data acquisition unit. **b**, Optical micrographs show *C. elegans* flowing from the microfluidic layer (blue), into the recording chamber and immobilized against a nano-SPEAR. **c**, False colored scanning electron micrograph of a representative nano-SPEAR suspended midway between a layer of silicon (gray) and a layer of photoresist (red) that forms a recording chamber for immobilized *C. elegans*. (inset) zoom-in of the nano-SPEAR shows a 100 nm Pt layer (light gray) on top of a 300 nm thick insulating layer of silicon oxide (blue). **d**, Representative confocal image of a worm expressing YFP in the body-wall muscles immobilized against a nano-SPEAR. The nano-SPEAR (white box), much smaller than an individual body wall muscle, is enveloped by the muscle cell. **e**, Locomotive phenotyping of WT animals on Nematode Growth Media shows that worms that have been immobilized in a nano-SPEAR device remain intact and have no significant difference in crawling velocities from control animals (n = 10 for each cohort, error represents the standard error, ns = not significant via unpaired, two-sided Student’s t-test). The velocities for each group are within the bounds of with previously reported values^25^.

To create a platform for scalable, cellular-resolution electrophysiology in intact small animals, we invented a new electrode geometry that we call **nano**scale **s**us**p**ended **e**lectrode **ar**rays (nano-SPEARs) (Fig. 1a–d; Supplementary Fig. 1). These electrodes—designed to be smaller than an individual cell—are suspended above the surface of a microfluidic chamber. This geometry allows the electrodes to press tightly against animals confined inside the chamber, providing a strong electrical coupling between the nano-SPEAR and the adjacent cell or cells. We specifically designed our first-generation nano-SPEAR devices to probe the body-wall muscles in the 1 mm-long worm, *C. elegans* (Fig. 1b–d). These 95 muscle cells, roughly 100 μm in length, line nearly the entire worm body-wall. The proximity of these muscles to the outer cuticle and their repeated pattern along the worm make them a good target cell for testing our devices. We found that when we immobilized *C. elegans* inside the microfluidic chamber, individual muscle cells wrap around a nano-SPEAR that we manufactured to be roughly 25 times smaller than a single muscle cell (4 μm wide, 3 μm long, 100 nm thick) (Fig. 1d). Furthermore, animals remain intact after being immobilized against a nano-SPEAR and resume normal locomotion after removal from the device (Fig. 1e, Movie 1).

The concept of nano-SPEAR electrophysiology was inspired by recent experiments showing that high-aspect-ratio nano- and micro-structures brought into contact with cultured cells can develop an electrical coupling sufficient to record APs^26–31^. Based on these results we believed that nano-SPEARs would provide a less-invasive and scalable alternative to patch-clamp electrophysiology in small organisms like *C. elegans*.

Using thin-film deposition procedures and electron beam- or photo-lithography, we can create electrodes less than 200 nm thick with a width that can be tuned from less than 200 nm to more than 5 microns (Fig. 1c, Supplementary Fig. 2). By precisely tuning the geometry of the electrode we can ensure that the electrode is much smaller than the target cell, which could be as small as 2–4 μm in the case of *C. elegans* neurons^2^. Additionally we can fabricate nano-SPEARs on both silicon and glass substrates; the latter giving us the ability to make nano-SPEAR measurements while simultaneously performing fluorescence microscopy using an inverted microscope (Supplementary Fig. 1, Methods).

**Figure 2:**
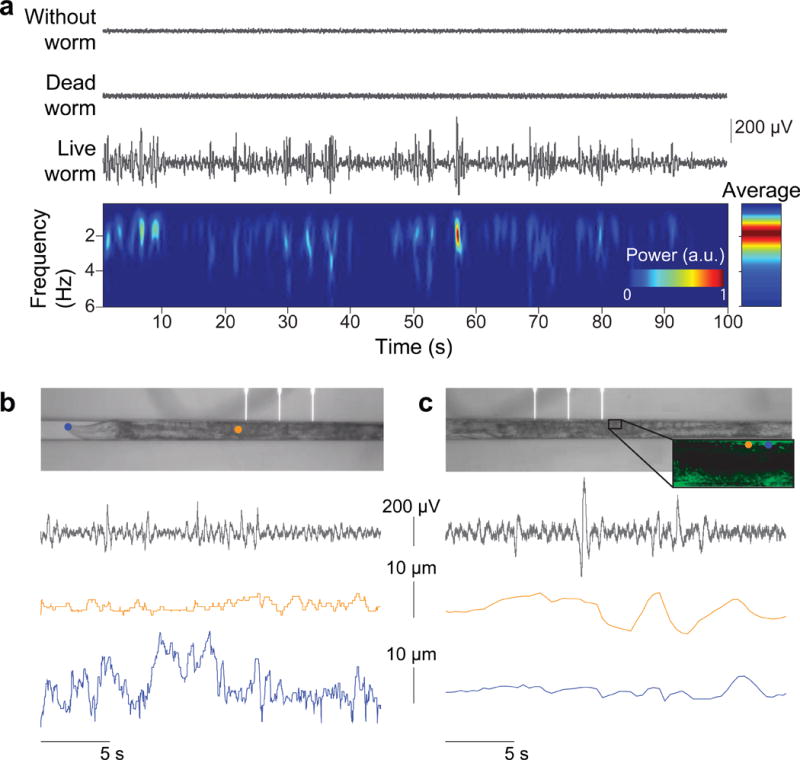
nano-SPEAR recordings are due to animal electrophysiology **a,** Traces recorded from nano-SPEARs on a glass substrate. (top) Nano-SPEAR measurement with no worm in the recording chamber. (center) Recording from a dead worm immobilized on a nano-SPEAR. (bottom) The trace and corresponding spectrogram show a dramatic change in the amplitude and frequency of measurements when a worm is immobilized against a nano-SPEAR. The spectrogram shows that spiking activity resides primarily in the 1**–**5 Hz frequency band. All traces have been bandpass filtered with cutoff frequencies of 1 and 100 Hz. **b**, Representative nano-SPEAR recordings (gray) show no apparent correlation with simultaneously recorded displacement of the body (orange) or the head (blue). **c**, Likewise, representative nano-SPEAR recordings (gray) show no apparent correlation with simultaneously measured muscle-cell micro movement (orange and blue). Detailed analysis of simultaneously recorded movement and nano-SPEAR data can be found in Supplementary Fig. 3.

Our initial experiments with wild type (WT) *C. elegans* suggest that nano-SPEARs indeed record electrical activity from cells within an intact worm. When we immobilized WT day-1 adult worms against a nano-SPEAR we observed an immediate increase in the variance of the voltage waveform and an appearance of putative “spikes” (Fig. 2a). This signal change was not seen when euthanized animals were brought into contact with the electrode (Fig. 2a). To ensure that the recordings are electrophysiological in nature and not due to worm movement, we recorded activity while simultaneously imaging the worm body and muscle cells (Fig. 2b–c, Movies 2–3). We found that neither movement from the worm’s head nor body correlated with our measurements (Fig. 2b, Supplementary Fig. 3, Movie 2). In addition, micro-movements of the muscle cells themselves also did not correlate with our recordings (Fig. 2c, Supplementary Fig. 3, Movie 3). These observations combined with confocal images showing nano-SPEARs engulfed by body-wall muscles (Fig. 1d) suggest that nano-SPEARs record extracellular electrophysiological activity from the body-wall muscles.

## Recording *C. elegans* muscle activity

**Figure 3:**
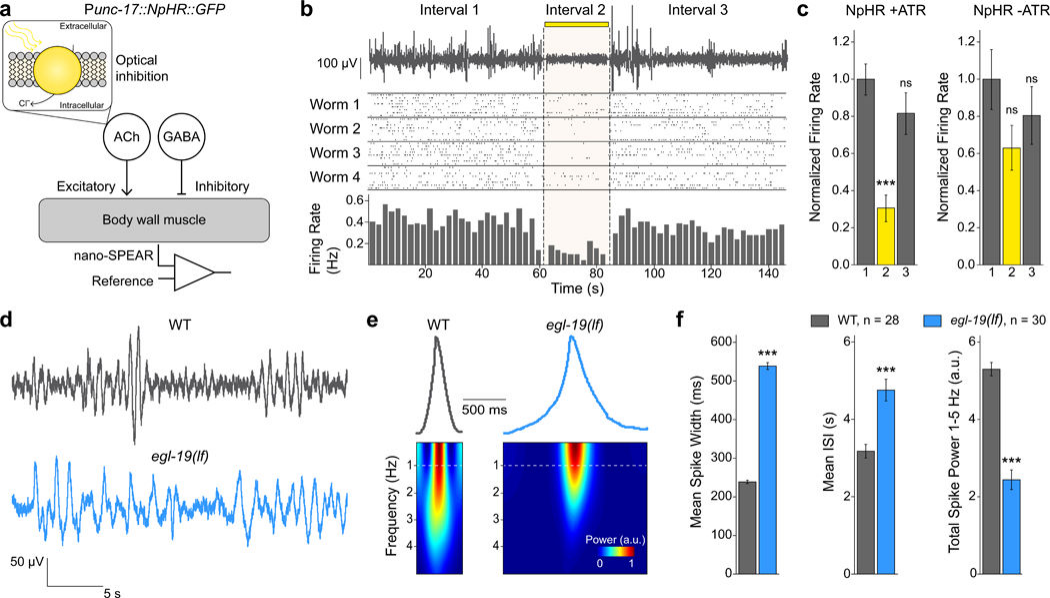
nano-SPEARs record electrophysiological activity from *C. elegans* body-wall muscles **a,** An illustration of the *C. elegans* body-wall neuromuscular junction with the light-gated chloride pump, NpHR, expressed in excitatory motor neurons under the *unc-17* promoter. **b**, Worms expressing NpHR show a reduction in firing rate under yellow illumination (Interval 2). Top trace shows a representative nano-SPEAR recording (bandpass filtered between 1 and 100 Hz). Yellow bar denotes the time period of yellow light (540**–**600 nm) illumination. Raster plot (center) shows spike times recorded from 4 different worms each tested 8 times. The histogram shows a drop in spike frequency during illumination. **c**, Mean firing rates for test worms raised with ATR (NpHR +ATR) show a significant drop in firing rate during illumination. A small, but not statistically significant drop in the firing rate was observed in control animals (NpHR −ATR) during illumination, likely due to a weak endogenous photo-inhibition effect. (n = 4 for NpHR +ATR, 32 total trials; n = 5 for NpHR −ATR, 40 total trials; ***p < 0.001, ns = not significant, one-way ANOVA with a *post hoc* Bonferroni test compared to Interval 1, error bars are the standard error). **d**, Representative data from WT (gray) and *egl-19(lf)* (blue) mutant worms. Traces are bandpass filtered with cutoff frequencies of 0.3 and 100 Hz. **e**, Average waveforms for each strain. The spectrogram is calculated from the mean waveform. Qualitative differences can be seen in the waveform width and the total power in the frequency band above 1 Hz. **f**, As expected from previously reported patch-clamp measurements*, egl-19(lf)* animals have a longer spike width and ISI. Longer *egl-19(lf)* waveforms also lead to less power in the 1**–**5 Hz frequency domain in the spectrograms from (e) (n = 28 for WT and 30 for *egl-19(lf)*; ***p < 0.001 unpaired, two-sided Student’s t-test, error bars represent the standard error).

Additional experiments using optogenetic inhibition suggest that the majority of voltage spikes recoded in the 1–5 Hz frequency band correspond to electrical activity of the body-wall muscles. For example, we recorded from a transgenic strain expressing the inhibitory light-gated chloride pump halorhodopsin (NpHR) in the excitatory motor neurons (*unc-17* promoter, Fig. 3a). When we illuminated animals with yellow light (540–600 nm) we recorded a dramatic decrease in the firing rate (Fig. 3b–c, p < 0.001). The firing rate then returned to pre-stimulus levels when we removed the optical stimulus (Fig. 3b–c). We observed no light-dependent change in electrical activity in a group of control worms raised in the absence of *all-trans* retinal (ATR, a small molecule required for functional NpHR^32^) (Fig. 3c). These results indicate that the spikes recorded by nano-SPEARs primarily represent muscle-cell activity that is driven by excitatory motor neurons, consistent with results from patch-clamp recordings^33^. This conclusion is also supported by experiments using transgenic animals expressing NpHR directly in the muscle cells (*myo-3* promoter, Supplementary Fig. 4a). While direct inhibition of muscle cells is less effective at arresting animal locomotion (likely due to the fact that the muscles continue to receive excitatory input, see Methods), we still measured a significant drop in the firing rate upon yellow-light illumination (Supplementary Fig. 4b, p < 0.05). Thus muscle-cell activity is a major contributor to the spikes observed in nano-SPEAR recordings.

As further confirmation that nano-SPEARs record electrophysiological activity from the body-wall muscles we found that *egl-19(n582)* and *shk-1(ok1581)* loss-of-function (lf) mutant worms show longer putative spike waveforms as compared to WT worms, which is consistent with previously reported patch-clamp electrophysiology data for body-wall muscle APs^7,33,34^ (Fig. 3d–f, Supplementary Fig. 5). In addition, *egl-19(lf)* have been shown to have a longer inter-spike-interval (ISI) as compared to WT animals^33,34^. When we analyzed our nano-SPEARs recordings from *egl-19(lf)* animals we found that the mean spike width is more than 2 times longer than the mean WT width and the inter-spike interval is 50% greater than WT animals (p < 0.001, Fig. 3e–f). Similarly we found that nano-SPEAR recordings from *shk-1(lf)* animals showed approximately 15% longer waveforms as compared to WT (p < 0.001, Supplementary Fig. 5). For both *egl-19(lf)* and *shk-1(lf)*, we found that these phenotypes do not depend on a particular choice of the spike-detection threshold (see Methods). Additionally, when comparing the spectrograms of the elongated *egl-19(lf)* and *shk-1(lf)* waveforms to WT, we observed a significant reduction in power in 1–5 Hz frequency band, indicating that a frequency domain analysis may also help phenotype mutant strains (Fig. 3e–f, Supplementary Fig 5).

Together, the results of our optogenetic experiments and electrical recordings from mutant strains suggest that muscle-cell activity is the primary source of the spikes observed in nano-SPEAR recordings. It should also be noted that because optogenetic inhibition does not completely eliminate muscle-cell activity, more work is needed to determine if synaptic and neuronal activity also contributes to spikes or if the remnant spiking activity is due to the muscle-cell activity that persists during yellow-light illumination. Future work will also help determine if spiking activity originates solely from individual muscle-cell APs; however, the small size of the electrode ensures that it is not in contact with* more than a few muscle cells at the same time, suggesting that activity from only one or two cells dominate the recordings. It should also be noted that the tapered linear channels used for worm immobilization likely reduce muscle cell activity, which is known to depend upon posture and proprioception^35^. Thus, comparisons between immobilized worms may not represent muscle activity in freely moving animals, but is nevertheless sufficient to identify phenotypic differences between mutant strains.

Because nano-SPEAR recordings do not require dissections we recorded from nearly 60 worms over the course of four days (n = 28 WT, n = 30 *egl-19(lf)* in Fig. 3d–f), roughly twice the throughput of patch-clamp techniques (n = ~6–7/day)^6^. To determine the minimum sample size necessary to accurately phenotype these mutant strains, we selected random subsets of worms from the full dataset and calculated the spike width and ISI. We found that on average a sample size of only 6 worms was sufficient to quantify these metrics with less than a 6% error (Supplementary Fig. 6).

**Figure 4:**
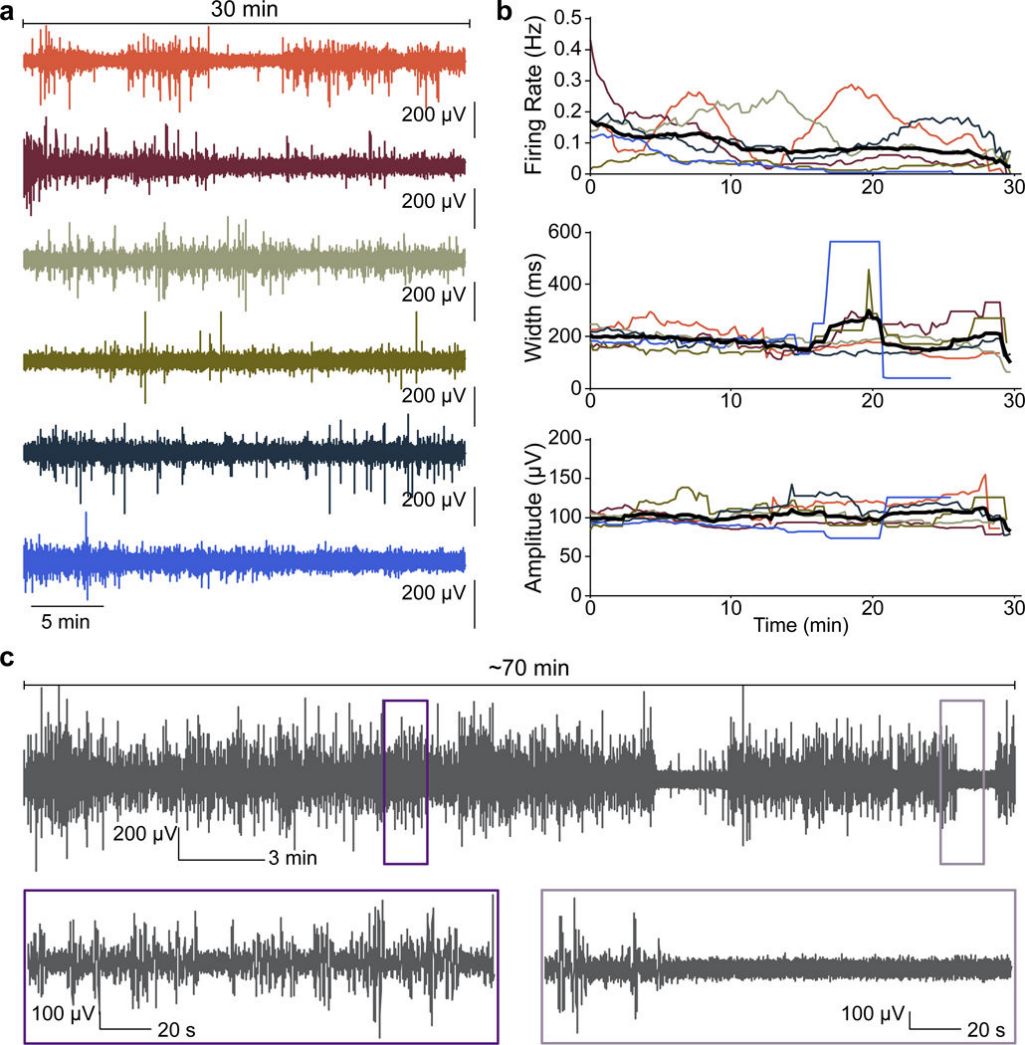
nano-SPEARs record continuously for tens of minutes **a,** Continuous 30 min recordings from six WT animals (traces are bandpass filtered between 1 and 100 Hz). **b**, We quantified the firing rate (top), spike width (middle) and spike amplitude (bottom) for each animal with respect to time. Black lines indicate the mean. The firing rate varies significantly from worm to worm with most animals showing a decrease in firing rate over time; however, one animal displayed periods of quiescence lasting for several minutes (orange traces). On the other hand, the spike width and spike amplitude remain relatively constant throughout the recording; however, when the firing rate diminishes to near zero (blue trace), the width and amplitude become highly variable because there are a small number of spikes that contribute to the ensemble average. **c**, A 70-minute-long continuous recording from a WT animal (traces are bandpass filtered between 1 and 100 Hz). Similar to (b), this animal showed distinct periods of quiescence during the second half of the recording, indicated by a clear drop in the spike rate. Insets are 3 min intervals that show normal spiking activity (left) and the abrupt transition to quiescence (right).

In addition to increasing throughput, recording from intact animals allows us to record from the same animal on consecutive days (Supplementary Fig. 7), raising the exciting prospect of measuring how age, disease progression, or drug treatment affect electrophysiology over the lifetime of individual animals. Additionally, we can consistently perform longer measurements than conventional methods (Fig. 4). We have reliably achieved 30 min recordings (Fig. 4a–b, n = 6), and in some cases recorded for more than 1 hr (Fig. 4, n = 2). The major factor limiting recording times is the fact that worms eventually crawl back along the tapered channel away from the nano-SPEARS; however, improvements in the immobilization strategy could extend the maximum recording time. While future work will help determine the ultimate factor limiting the length of nano-SPEAR recordings, these long recordings may result in the discovery of previously unknown *C. elegans* phenotypes and behaviors, such as a distinct period of quiescence exhibited by some WT adults (Fig. 4).

## Versatility of nano-SPEAR recording technology

The fact that integrated nano-SPEAR devices are manufactured using semiconductor microfabrication technology allows us to easily reconfigure the chip layout to accommodate differing *C. elegans* postures and even interrogate other animal species. For example, we can record from multiple sites along a single worm and also record from worms immobilized with a curved posture that more closely resembles their natural body position (Supplementary Fig. 8). Furthermore, nano-SPEARs are not limited to nematode-like organisms. In fact, we can dramatically alter the geometry of the recording chamber to record from the freshwater cnidarian *Hydra littoralis*, which has distinctly different neuroanatomy^36^ (Supplementary Fig. 9). We envision that the ability to tailor microchips for specific experiments in a variety of model organisms will allow for a new paradigm for integrated small animal electrophysiology with broad impact across many neurobiology disciplines.

In addition to reconfiguring recording chambers for different species, we can also create arrays of chambers that, when paired with the appropriate microfluidic channels, can record from multiple animals simultaneously (Supplementary Fig. 10a–c). In a configuration designed for immobilizing up to four *C. elegans* we performed 10 experiments (for a maximum possible 40 worm recordings). In these experiments, we immobilized a total of 30 animals and detected spikes in 24 (Supplementary Fig. 10). These trials were conducted over the course of only 1.5 hr. Thus, we achieved a throughput of 16 animals/hr—a significant improvement over conventional patch-clamping techniques (~1 animal/hr). Improving and automating the worm loading process (see Methods) can even further increase throughput, and provides the first clear path towards high-throughput electrophysiology at the *C. elegans* body-wall muscles at rates in excess of 100 animals/day.

## Phenotyping neurological disease models

The high-throughput potential of our nano-SPEAR technology would be particularly powerful for studying the synapses at the worm neuromuscular junction. These synapses have a conserved structure throughout the body-wall^37^ and share many genetic similarities to mammalian synapses, making them a good model for studying synaptic transmission^5,8,38^ and neurodegenerative diseases^39–41^. In fact, many human neurodegenerative diseases can be modeled at the *C. elegans* neuromuscular junction (NMJ) including Alzheimer’s disease, PD and ALS^39,42^. High-throughput electrophysiology would enable large-scale screens for drugs that effectively treat *C. elegans* models for neurological disease and could lead to improved treatments for human disorders^39,40,43^.

To demonstrate how nano-SPEARs can facilitate the study of neurological diseases, we selected *C. elegans* models for PD and ALS^44,45^. These diseases are modeled by heterologous expression of human genes implicated in disease pathogenesis, namely, alpha-synuclein (α-syn) for PD and G85R Cu, Zn-superoxide dismutase-1 (SOD1) for ALS. While reduced animal locomotion and cell loss have been shown in these disease models^44,45^, the electrophysiological phenotypes remain unknown, likely due to the difficulty of performing electrophysiology.

**Figure 5:**
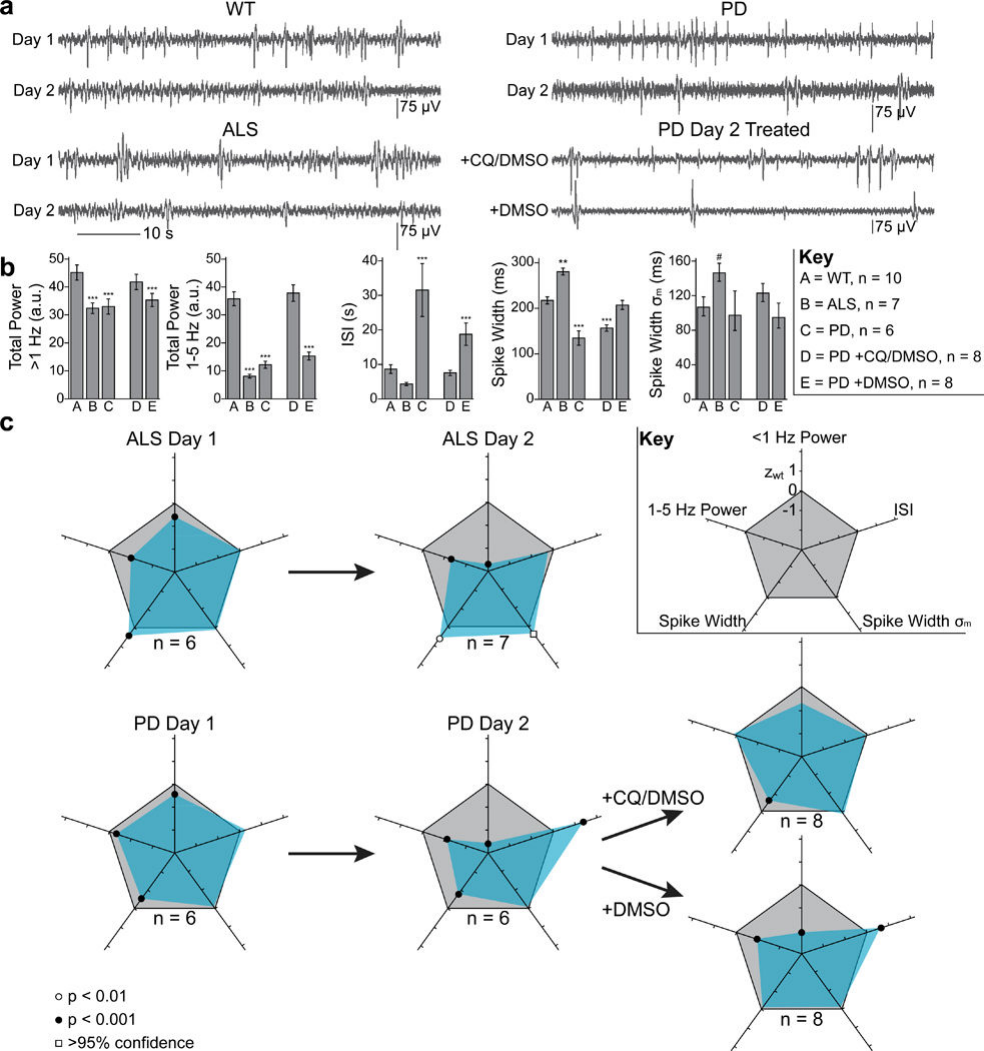
nano-SPEARs reveal phenotypes for neurodegenerative disease models **a,** Characteristic traces for WT and all disease models tested (bandpass filtered between 1 and 100 Hz). Day-1 and day-2 measurements represent distinct cohorts of animals selected randomly from a synchronized population on consecutive days. **b**, Quantified electrophysiology metrics (see Methods) for day-2 adult worms show differences in spike statistics between disease models and age-matched WT controls (**p < 0.01, ***p < 0.001; Kruskal-Wallis with a *post-hoc* Dunn-Sidak test with respect to WT for power metrics, one-way ANOVA with a *post-hoc* Bonferroni test with respect to WT for ISI and spike width; # > 95% confidence interval for WT). PD worms treated with CQ (+CQ/DMSO) show a full rescue of the power metrics and mean ISI, which is not observed in control worms (+DMSO) (see Methods). Error bars represent the standard error, or 95% confidence intervals in the case of Spike Width σ_m_. **c**, Phenotypic maps show a geometric visualization of the data in (b). Cyan polygons are created by connecting the z-score with respect to WT (z_wt_) on five axes representing different metrics (see Key). A phenotypic map for a WT animal creates the gray pentagon. ALS and PD worms show distinct age progressive phenotypes. When PD larvae were raised in the drug CQ (+CQ/DMSO), day-2 adults showed nearly complete rescue of the WT five-dimensional phenotype.

To identify how neuromuscular activity in PD and ALS disease models differs from healthy WT worms, we compared nano-SPEAR recordings in day-1 and day-2 adult worms to age-matched WT animals (Fig. 5a). Because day-2 animals showed more pronounced phenotypes, we compared several electrophysiological metrics in day-2 worms to see if we could better distinguish ALS and PD worms using a high-dimensional phenotype (Fig. 5b, see Methods).

While common phenotyping metrics such as paralysis and lifespan make PD and ALS worm models almost indistinguishable^44,45^, we found that using multiple electrophysiological metrics based on nano-SPEAR recordings allows us to clearly differentiate between the two disease models (Fig. 5b). To more clearly visualize the high-dimensional phenotypes we created phenotypic maps by plotting the z-score with respect to WT for each metric on a polar plot (Fig. 5c, see Methods). Most notably, we observed a dramatic increase in the inter-spike interval (ISI) for PD worms (~300%, p < 0.001) that was not observed in ALS worms. We also found a significant increase in the mean spike width of ALS worms (~30%, p < 0.001) while PD animals exhibited shorter waveforms (~40%, p < 0.001). We also measured the variability in the spike width (σ_m_) and discovered that the ALS distribution of spikes increased in variability compared to WT. To our knowledge, this data represents the first electrophysiological phenotypes for PD and ALS worm models.

While more work is needed to understand why ALS and PD models show such different electrophysiological phenotypes, one explanation is the fact that each disease model expresses the respective disease-related gene in a different group of cells: ALS is modeled in neurons while PD is modeled in the body-wall muscle itself^44,45^. This hypothesis suggests that synaptic input in ALS worms may reduce overall excitatory input to the body-wall muscle without affecting their relative health and mean ISI. In PD worms, however, degradation from α-syn of the body-wall muscles themselves may make them less capable of firing many successive spikes leading to a larger mean ISI.

Because nano-SPEARs have the ability to phenotype disease states in ALS and PD, we hypothesized they could also measure the efficacy of drugs for treating electrophysiological deficits. Indeed, when we measured day-2 adult PD worms that were treated with the known neuroprotective drug clioquinol (CQ)^46^, we discovered that the electrophysiological symptoms associated with this disease model were dramatically improved compared to a control group (Fig. 5a–c, see Methods). Notably, the ISI of treated animals was fully improved, supporting the hypothesis that α-syn inclusions inhibit the cell’s ability to spike with a regular rate. However, we did not see an improvement in the spike width of treated animals, which further emphasizes the fact that multiple metrics must be used to evaluate drug efficacy. Finally, in control animals we surprisingly measured an improvement of the width of waveforms. This supports previous reports that DMSO alone can affect *C. elegans* neurodegenerative models^47^. The phenotypic maps show that, compared to the control worms, treated PD worms have an electrophysiological profile that more closely matches WT (Fig. 5c). Overall, our ability to phenotype mutant strains and measure drug efficacy, shows that nano-SPEAR electrophysiology can facilitate drug screening and drug development using *C. elegans* as a model organism.

## Discussion

In summary, we found that nano-SPEARs provide a platform for scalable and versatile electrophysiology in intact small organisms. We have shown that the nano-SPEARs presented here capture activity from *C. elegans* body-wall muscles, and that these measurements allow us to phenotype neurological disease models and test for drug efficacy. Additionally, we have shown that nano-SPEARs are not limited to recordings from *C. elegans*, but can record activity in other small organisms like *Hydra*. The new capabilities of our system, such as long-term recordings (Fig. 4) and the increased throughput (Supplementary Fig. 10) opens the door to highly versatile electrophysiological phenotyping of these common model organisms. Although electrical measurements of *C. elegans* body-wall muscles provide rich data for studying NMJ activity and disease models, future work with smaller nano-SPEARs with widths on the order of ~100 nm (Supplementary Fig. 2) combined with more precise animal manipulation may enable intracellular access to the body-wall muscles and even recordings from neurons. A clear goal for future work is to develop nano-SPEARs that can perform voltage and current clamp experiments to measure ion channel behavior and postsynaptic potentials. We also envision that future work will combine the high-throughput, versatile electrophysiology of nano-SPEARs with other high-throughput assays^48^ to help uncover fundamental relationships between genetics, electrophysiology, and behavior using small model organisms.

## Methods

### *C. elegans* strains and culture

All *C. elegans* strains obtained from the CGC were: N2 as wild-type (WT); ZX299 *zxEx22*[*myo-3p∷ChR2(H134R)∷YFP + lin-15(+)*]; CB5600 *ccIs4251 [(pSAK2) myo-3p∷GFP∷LacZ∷NLS + (pSAK4) myo-3p∷mitochondrial GFP + dpy-20(+)*] I; EG4813 (*unc-17p∷NpHR∷GFP*); MT1212 *egl-19(lf)(n582) IV*; RB1392 *shk-1(lf)(ok1581)* outcrossed with WT 6X; and NL5901 *pkIs2386* [*unc-54p∷α-syn∷YFP + unc-119(+)*].*egl-19(lf)* encodes L-type voltage-gated Ca^2+^ channels^1,2^ and *shk-1(lf)* encodes voltage-gated K^+^ channels expressed in muscles^3,1^. The strain YX9 [*myo-3p∷NpHR∷ECFP;lin-15(+)*], 3X outcrossed was obtained from Anthony Fouad. The disease models used were NL5901 (PD) and the ALS strain was obtained from Jiou Wang from the work conducted in Wang, 2009 *et al.*^4^. Specifically these disease models express alpha-synuclein (α-syn) fused to YFP in the body-wall muscles (PD worms)^5^ and the G85R mutation of SOD1 fused to YFP in all neurons (ALS worms)^4^. All strains were maintained at approximately 21°C on nematode growth medium (NGM) seeded with OP50, unless stated otherwise.

### *Hydra* strains and culture

*Hydra littoralis* was obtained from the Carolina Biological Supply Company. The *Hydra* were cultured at approximately 21°C in *Hydra* medium in a dark environment and fed freshly hatched *Artemia naupli* every other day. *Hydra* were starved for at least 24 hours before experiment to facilitate microfluidic manipulation.

### Device fabrication

Silicon (Si) and glass devices were fabricated using conventional micro- and nano-fabrication techniques^6^. For Si, Pt was sputtered onto a thermal oxide grown on a highly-doped Si wafer (NOVA Electronic Materials) with an electron-beam or photo-lithography defined electrode pattern. During recordings, the Si wafer is grounded to reduce noise. Al_2_O_3_ is grown via atomic layer deposition to insulate and protect the top of the Pt. Photolithography with the thick photoresist KMPR-1025 (MicroChem) then defines the recording chamber. The photomask is aligned such that the tips of the Pt electrodes protrude from underneath the KMPR. We suspended the electrode tips by reactive ion etching (RIE) the exposed oxide and Si substrate.

Fabrication on a glass substrate (University Wafer) has the advantage of reducing the parasitic capacitance and allowing for high-resolution imaging through the glass substrate using an inverted microscope. Instead of Si, Pt electrodes are deposited onto a KMPR layer. A second layer of KMPR is deposited on top of the Pt electrodes and the recording chambers are defined using photolithography. Next, we use a RIE to etch the bottom KMPR layer and suspend the electrodes. Due to KMPR being a poor water barrier, devices fabricated on a glass substrate typically have a short lifetime (1–3 days) once in contact with buffer solution. This timeframe can be increased to the order of weeks by coating the entire wafer in ~100 nm of Parylene C. Focused ion beam milling can then be used to expose the electrode tips. This additional process was used data in Supplementary Fig. 4, Supplementary Fig. 10 and Fig. 4. For more details on fabrication, see Supplementary Fig. 1.

The completed wafers are connected to a PDMS (Sylgard) microfluidic layer that is approximately 4-mm thick and molded from SU-8 2050 (MicroChem). A custom acrylic enclosure is used to clamp the PDMS to the wafer and prevent fluid leakage. A single recording chamber and worm trap have a combined fluidic volume of approximately 0.7 μL. We used conductive epoxy to bond electrical leads to on-chip Pt pads. The average nano-SPEAR impedance is 1.5 MΩ at 1000 Hz.

### *C. elegans* electrophysiology

For each worm recording, we picked an individual worm from NGM and placed it an open syringe cap filled with M9 buffer. Tubing connected to the syringe cap leads to the microfluidic device and nano-SPEAR microchip. Suction applied to an opposing syringe brings the worm into the recording chamber. As previously seen in microfluidic devices^7^ and due to a chamber height (~60 μm) just large enough for adults, worms naturally undergo crawling motion when traveling down the tapering channel to the worm trap, ensuring that in most cases the body-wall muscles are in the proper orientation with respect to the nano-SPEAR. Because of this, we achieved an 80% success rate for recordings (i.e. we detected spikes in 24 out of 30 immobilized animals, see data in Supplementary Fig. 10). When performing imaging we saw no significant correlation between the signal-to-noise ratio of recordings and the positioning of muscle cells relative to the electrode. After recordings, we flushed worms off-chip and removed them from the tubing. Unless stated otherwise, we analyzed only the first five minutes of data from each worm. All data was obtained with an Intan Technologies RHD2216 bipolar input amplifier at a sampling rate of 10 KHz, low frequency cutoff and DSP filter of 0.1 Hz and high frequency cutoff of 7.5 KHz. A nano-SPEAR not in contact with worms was used as a reference and a low-impedance Ag/AgCl electrode in fluidic contact with the worm trap was used as ground.

### Locomotive phenotyping

We immobilized 10 day-1 WT animals in a nano-SPEAR recording chamber for 5 min. Following recordings, worms were flushed from the device and onto NGM seeded with OP50. Control worms (n = 10) were continually kept on seeded NGM. Using previously established tracking software^8,9^, we measured animal locomotion for 2 min on a fresh, 10 cm NGM plate with a thin, even layer of OP50 spread across the surface. Tracking occurred within 4 hours of recordings. These experiments took place on a single day.

### Spike detection and analysis

The algorithm we developed to choose *C. elegans* spikes first involves using a high-order bandpass filter to reveal spiking activity. For phenotyping all disease models, the low- and high-cutoff frequencies used were 1 and 100 Hz, respectively. When comparing the spike waveforms of WT, *shk-1(lf)*, and *egl-19(lf)* we used cutoff frequencies of 0.3 and 100 Hz. Before and after each experiment, we recorded several minutes of data from with no animals on the nano-SPEARs to determine the baseline RMS noise. We used a threshold of six-times the background RMS value for detecting spikes in the filtered data, but the reported trends between WT and other strains were constant when this threshold was increased. Both positive and negative waveforms are detected in the recordings, likely due to worm micro-movement and small changes in cell-electrode coupling. Therefore, we searched for both negative and positive spikes when analyzing data. To avoid any potential artifacts of the filtering process we used raw data when comparing waveforms (with spike timing identified using the filtered data as described above). To form average waveforms, all detected spikes from each strain were made positive, scaled to the peak value, and time aligned to 75% of the max value on the rising edge. The reported spike widths are the full-width, half-max values. Unless otherwise stated, all error bars represent the standard error.

### Spectral analysis

We calculated the spectrogram from the raw data from each worm with a sliding window, multi-taper spectrogram function from Chronux (http://chronux.org/). Averaging all spectrogram frequency bins across time produced the power spectrum for a worm with a standard error for each frequency data point. We then averaged each individual power spectra to produce an average power spectrum for the strain (using standard error propagation). For comparison between strains, we normalized the spectra by dividing by the maximum power.

### Movement and electrophysiology recordings

Nano-SPEAR recordings were conducted while simultaneously performing either bright-field or fluorescence imaging to measure head/body or muscle movement, respectively. WT animals were used for bright-field and the ZX299 (grown in the absence of ATR) for fluorescence imaging. The CB5600 strain was used for Movie 3. The qualitative (Fig. 2) and quantitative (Supplementary Fig. 3) data shown are representative from two animals; however, we have found similar results from other experiments. To eliminate photocurrent artifacts during laser scanning confocal microscopy, we tracked the movements of a muscle cell near the electrode rather than the cell directly against the nano-SPEAR. To account for any offset in the timing of the electrical and optical data, we measured the maximum of the cross-correlogram rather than the cross-correlation value at zero offset. To determine if the peak in the cross-correlogram represented statistically significant correlation between nano-SPEAR recordings (μV) and worm movement (μm), we separated each data set into a series of 2–10 s intervals that could be shuffled to remove any potential correlation. For bright-field imaging, we used a 2 s interval that shifts with a step size of 0.2 s until reaching the end of the data sets. For fluorescence, we used a 10 s interval that shifts with a step size of 1 s until reaching the end of the data sets. To remove any potential correlation between the nano-SPEAR recordings and movement data we independently shuffled the intervals of each data set. Calculating the correlograms, then extracting the maximum of the correlograms in each window of these shuffled data sets yields a distribution of correlation values that represent uncorrelated data. These data became the histogram of peak correlogram values for uncorrelated data. We then formed a similar distribution, based on the peak correlogram values of the unshuffled (e.g. time-aligned) data. We expect these distributions to be significantly different if movement is correlated with nano-SPEAR measurements; however, we found no such differences indicating that nano-SPEAR recordings are not correlated with animal movement.

### Optogenetics

EG4813 and YX9 worms were raised in the dark on NGM plates seeded with OP50 and transferred to NGM plates seeded with 100 μL of OP50 supplemented with 2 μL of *all-trans* retinal (ATR) approximately 48 hr before experiments. Control worms were never in the presence of ATR and were also raised in the dark. Worms were imaged with white light filtered through a red transparency when entering the chamber, and yellow illumination (540–600 nm at 10 mW/mm^2^) was delivered with an X-cite XLED1.

EG481 animals under yellow illumination showed prolonged paralysis when crawling on agar. Therefore, we chose trials that consisted of a 60 s period of no illumination (Interval 1), a 20 s period of yellow illumination with (Interval 2), and a final 60 s period of no illumination (Interval 3). Each worm was tested 8 times. For YX9 animals, we noticed yellow illumination of adult animals inhibited crawling on agar for only a few seconds. Thus, we chose intervals of 5 s of no illumination (Interval 1), 5 s of yellow light (Interval 2) and 5 s of no illumination (Interval 3). Each animal was tested 8 times. Presumably, paralysis of EG481 animals is more robust due to the continued release of GABA into the synaptic cleft when only excitatory motor neurons are inhibited. NpHR experiments were not performed in the *lite-1* background^10^. The reported data in Fig. 3 and Supplementary Fig. 4 were taken across four days, one day for each +ATR and −ATR group.

### *C. elegans* phenotyping

Synchronized populations were attained by standard bleaching methods. For all experiments, researchers were aware of the strains being tested. WT and other controls were done in no particular order and researchers recorded from only one strain of animals at a time. Slight differences from chip to chip (i.e. length of the electrodes, etc.) led to slight changes in the WT phenotype. To account for the variances in devices, mutant and transgenic worm were always compared to WT worms that were measured on the same device.

By detecting all spikes from each strain, we quantified the mean inter-spike interval (ISI) based on the time between spikes (the peak value defines the spike time). Also, by isolating each detected spike and measuring its full-width half-max, we calculated the mean width of a spike. The standard deviation of the distribution of spike widths for a strain is the metric σ_m_. For calculating power metrics, we summed the total power of the normalized spectrum (see “Spectral analysis” subsection) for a strain in the designated frequency bands (<1 Hz and 1–5 Hz). For ISI and width metrics, error bars represent the standard error. For σ_m_, error bars represent the 95% confidence intervals. For power metrics, error bars represent the propagated error from the power spectrum.

The WT and *egl-19(lf)* data was taken across four days, and the WT and *shk-1(lf)* data were taken on two separate days. The data displayed in Fig. 3 and Supplementary Fig. 5 show the full extent of these recording periods.

For transgenic disease models, we fabricated two sets of recording chambers with slightly different chamber widths to accommodate day-1 (~50 μm diameter) and day-2 (~60 μm diameter) adults and compared the recordings. The data for day-1 and day-2 animals represent two different groups of animals raised under the same conditions. To construct phenotypic maps, we created a polar plot such that each axis represents a single electrophysiology metric in units of the z-score compared to the WT value. The power metrics fell well beyond the z-score of the other metrics and were therefore scaled by a factor of 100 to make all z-scores comparable.

### Long-duration *C. elegans* recordings

Recordings were performed as other single-animal recordings, but were allowed to run for 30 minutes before being stopped. We found that the primary difficulty in long-duration recordings was worm immobilization. Over the course of tens of minutes, animals can move up to several hundred microns. In the recordings displayed in Fig. 4, animals drifted < 100 μm from the starting position. The quantified data in Fig. 4 represent animals from a single day of recordings, but extended recordings have been achieved with several animals on multiple microchips.

### Parallel *C. elegans* recordings

Like single-worm recordings, four animals were picked from NGM and suctioned into the device. For each trial, typically only 2–3 animals made it to the recording chambers successfully. This was due to a decrease in flow rate and poor suction as more and more chambers are filled. All recordings lasted 3 min before they were terminated. Worms were removed by flushing them back out the entry port. We began timing the experiment just before loading the first set of animals. Within 1.5 hr, 10 different sets of animals had been loaded and immobilized. The displayed data in Supplementary Fig. 10 represents the full extent of this 1.5 hr period, but similar throughput has been achieved on other occasions. Phenotypic maps were constructed identically to disease models (Fig. 5) to show that the differences between recording chambers produce negligible changes in the electrophysiological phenotype compared to the effects of the disease model.

### *Hydra* electrophysiology

Similar to *C. elegans*, an individual *Hydra* approximately 1–2 mm in size was pulled from *Hydra* media into clear tygon tubing attached to a syringe. Suction applied through microfluidic channels on the opposite end of the device brought the *Hydra* into the recording chamber (Supplementary Fig. 9). The microfluidic ceiling on the recording chamber flattened the *Hydra* and the narrow region near the electrodes immobilized the *Hydra* against the nano-SPEARS. Immobilization was stable throughout the experiment, with the exception of instances when extended body elongation led to a significant reduction in diameter of the *Hydra* body. The nano-SPEARs measured bursts of electrical activity during the animal contractions. These measurements resemble contraction bursts that are known to be associated with contraction^11^. The recording period was ended after 10 minutes. Upon completion of the experiment, *Hydra* was removed from the recording chamber through the syringe connected to the entry port. All data was obtained with an Intan Technologies RHD2132 unipolar input amplifier at a sampling rate of 10 KHz, low frequency cutoff and DSP filter of 0.1 Hz and high frequency cutoff of 7.5 KHz. The data in Supplementary Fig. 9 displays the full extent of all trials over the course of three days.

### Worm euthanizing

An adult WT worm was euthanized with M9 bath at 70°C for 1.5 min. All other recording conditions were identical to a typical experiment with live animals. The trace in Fig. 2 represents the entirety of the data collected.

### Clioquinol (CQ) treatment

CQ has been shown to protect *C. elegans* dopaminergic neurons from α-syn toxicity^12^. We hypothesized CQ would therefore protect body-wall muscles from α-syn toxicity and preserve WT electrophysiology. PD worms were synchronized using bleaching methods and the eggs were incubated in a 30 μM CQ, 1% DMSO (Sigma-Aldrich) in M9 solution for 24 hrs at room temperature. After the incubation period, the larvae were plated on NGM until reaching day-2 adulthood. In parallel with the treated worms, control worms underwent the same protocol but were never in the presence of CQ. The data from treated animals in Fig. 5 represent one test and one control group.

### Statistical analyses

All statistical methods used and subsequent p-values are reported in figure captions. For experiments in which t-tests or ANOVA tests were used, the data was assumed to be normally distributed and variance equality was checked using Levene’s test. If only two groups were being compared and the variance was found to be different, a Welch’s t-test was used rather than a Student’s t-test. In experiments that compared three or more groups, we found that only one data set, disease model phenotyping (Fig. 5), did not meet the equal variance requirements for ANOVA statistics. Significance overestimation is likely minimal because all groups have similar sample sizes in this experiment; however, to take into account the possibility of overestimating significance, we chose only p < 0.01 as significant for these metrics. To accommodate the possibility that some data may not be normally distributed, we also ran non-parametric Kruskal-Wallis tests for all experiments in which normality was assumed. In all but one case, the reported results remain significant. One experiment: the ISI metric when phenotyping day-2 disease models (Fig. 5) would not be significantly different unless normality is assumed. Thus, future work may be needed to fully interpret the ISI metric in immobilized disease models. To determine significance between frequency bands of power spectrums, we used a Kruskal-Wallis test with a *post hoc* Dunn-Sidak test.

### Data availability statement

The data that support the plots within this paper and other findings of this study are available from the corresponding author upon reasonable request.

## Supplementary Material

1
