# Supplementary material for: Scalable Electrophysiology in Intact Small Animals with Nanoscale Suspended Electrode Arrays
Source: Nat Nanotechnol. Author manuscript; Available in PMC 2017 Oct 17. (PMC5500410; doi:10.1038/nnano.2017.55)
Supplement: 1 [file NIHMS857483-supplement-1.pdf]

Scalable Electrophysiology in Intact Small Animals with Nanoscale Suspended Electrode Arrays

Daniel L. Gonzales, Krishna N. Badhiwala, Daniel G. Vercosa, Benjamin W. Avants, Zheng Liu, Weiwei Zhong, Jacob T. Robinson*

*Correspondence to: jtrobinson@rice.edu.

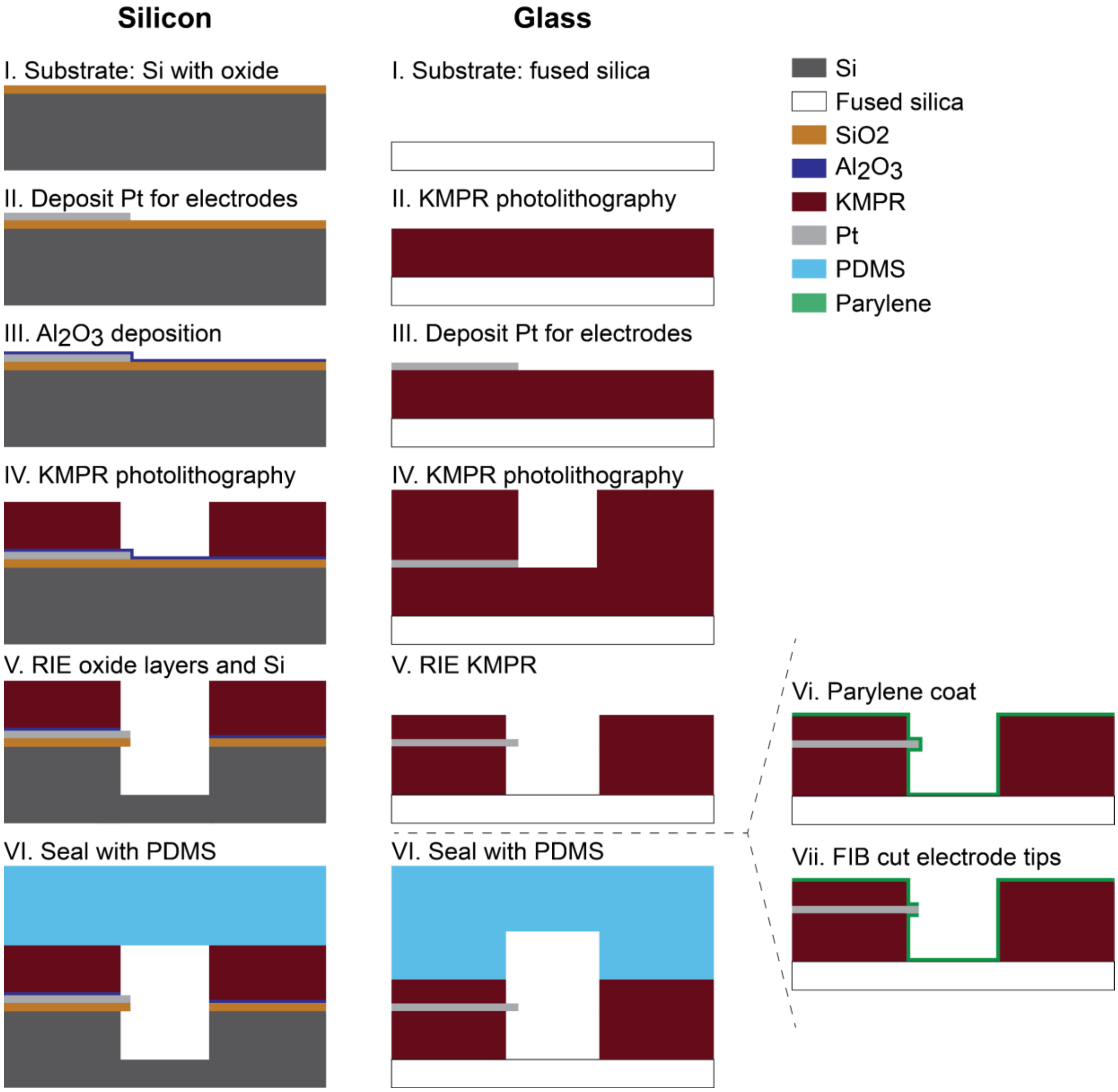

Supplementary Fig. 1 | Fabrication of suspended electrodes. (Left) (I) Fabrication on a Si substrate (gray, Nova Electronic Materials) with 300 nm of thermal oxide (yellow). (II) 300 nm of Pt is sputter coated on top of a patterned resist layer (photolithography (LOR 30B and S1818, MicroChem) or electron-beam lithography (950 PMMA C2, MicroChem)). Liftoff of the resist leaves behind the patterned electrodes (light grey). The mask used for lithography can be easily tailored to place the electrodes in the desired position or alter the electrode dimensions. (III) 20 nm of Al_2O_3 (purple) is deposited via atomic layer deposition to insulate the top and sides of the Pt. (IV) 30 µm of KMPR-1025 (MicroChem, red) is used to define the recording chamber via photolithography. The photomask used can be changed to create recording chambers that can accommodate varying sizes and orientations of worms or other animals. When performing the exposure, the photomask is carefully aligned such that approximately 3 µm of the electrode tips protrude from under the KMPR. (V) Reactive ion etching (RIE) removes the exposed Al_2O_3 and thermal oxide (CHF_3, C_4F_8, O_2). Another RIE process (cryogenic or Bosch) etches a total of 30 µm into the Si substrate (SF_6, O_2). This etch is tuned to be slightly isotropic and suspend the Pt

and underlying oxide. (VI) The final microchip is sealed with PDMS and the final chamber height is ~60 µm. Total fabrication time is approximately 15 hr.

(**Right**) (I) Fabrication on a fused silica (transparent, University Wafer). (II) KMPR-1025 is spun to coat the entire substrate with a 20 µm film that is then exposed to UV light to cross-link the polymer. (III) As before, 300 nm of Pt, and a liftoff procedure defines the electrodes. (IV) 30 µm of KMPR defines the recording chamber. In this case, the Pt is aligned to be fully embedded under KMPR. (V) RIE (SF_6, O_2) removes 20 µm of KMPR from both the top and bottom layers. The etch is slightly isotropic, which exposes and suspends the Pt. (Vi) Because KMPR is a poor water barrier, coating the device with ~100 nm of Parylene C is an optional process to increase device longevity. (Vii) Focused Ion Beam (FIB) milling is used to expose the Pt tips. These steps allow for microchips to be used on the order of weeks, rather than days for uncoated wafers. (VI) PDMS again seals the chamber; however, it is molded to fit the recording chamber for a final chamber height of ~60 µm. An inverted microscope can be used to image through the glass substrate. Total fabrication time is approximately 18 hr without parylene and 24 hrs with parylene.

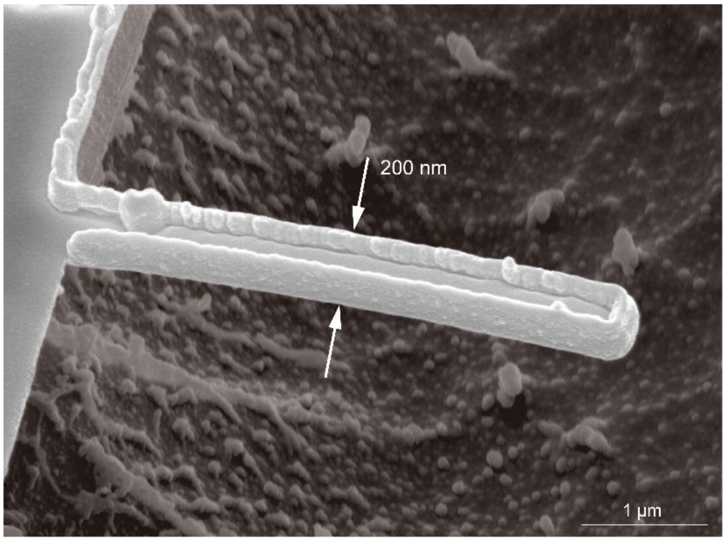

Supplementary Fig. 2 | 200 nm wide nano-SPEAR. Using electron-beam lithography, we fabricated 200 nm wide Pt nano-SPEARs. Even at this scale, nano-SPEARs can be suspended above a photoresist-based chamber (light red).

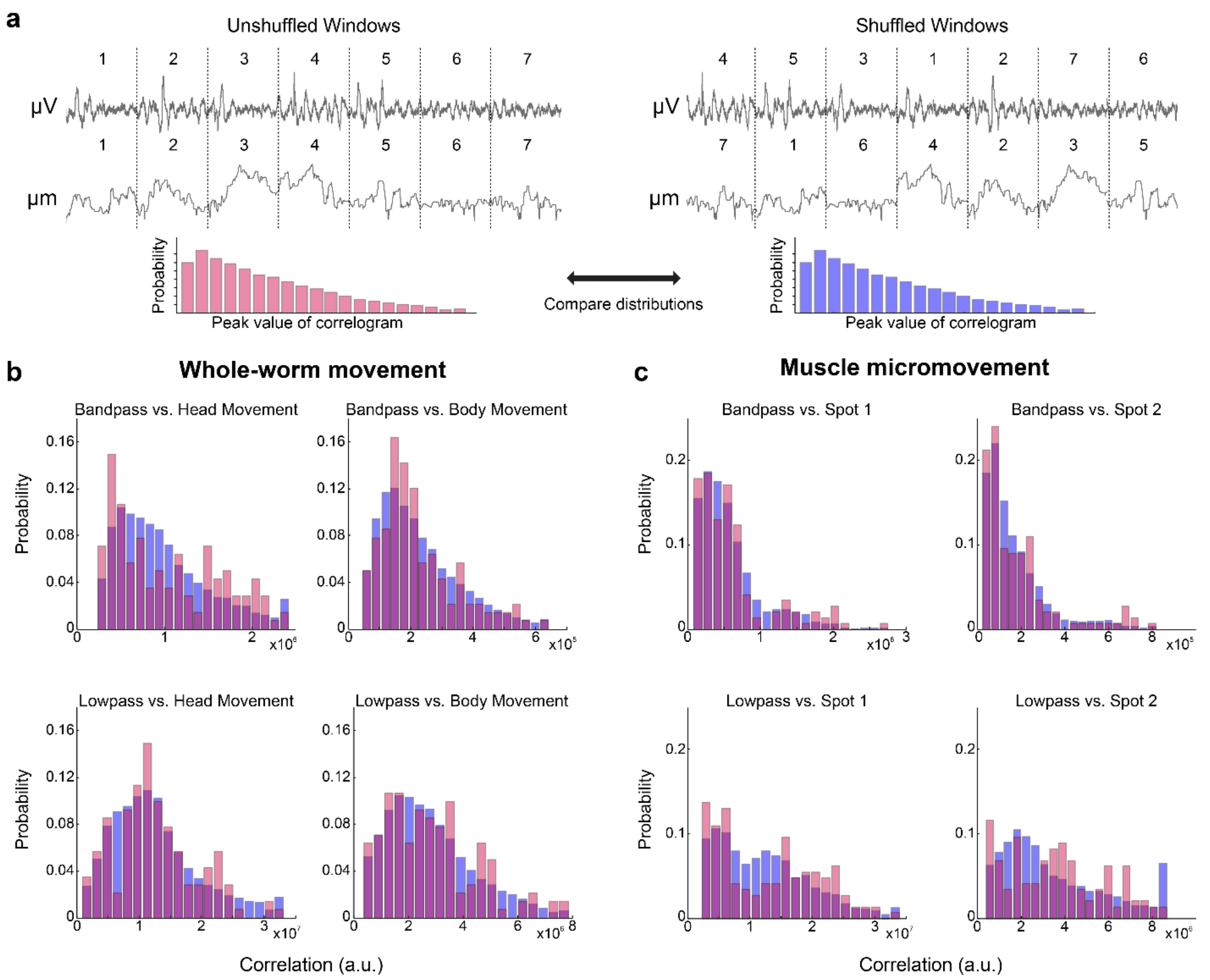

Supplementary Fig. 3 | Quantification of simultaneous worm movement and nano-SPEAR recordings. a, Graphic depicting the process of quantifying correlations between nano-SPEAR recordings (μV) and movement (μm). (left) The original data is separated into time intervals. Calculating the maximum value of the correlogram between the movement and nano-SPEAR data yields a distribution of correlation values for the unshuffled (e.g. time-aligned) data. (right) To test significance of the correlation values, the time intervals for both data sets are randomly shuffled. Calculating the maximum value of the correlogram in the shuffled data yields a distribution of correlation values that are known represent uncorrelated data. The original data can be considered correlated only when the shuffled and unshuffled distributions are significantly different. **b,** Correlation between electrophysiology and worm head and body movement. "Bandpass" and "Lowpass" refer to nano-SPEAR recordings that have been bandpass filtered (1 to 100 Hz) and lowpass filtered (<1 Hz), respectively. We performed this filtering to separate spiking activity (bandpass filtered data) from low frequency oscillations (lowpass filtered data). We initially hypothesized that low-frequency oscillations would correlate with worm movement; however, in all cases, we found nano-SPEAR distributions are not significantly different from worm head and body movement ($p > 0.05$, t-test comparing the means of each distribution). The data represent recordings from a single animal, but similar results were found in $n = 2$ animals. **c,** Correlation between electrophysiology and muscle movement. As in (**b**), we analyzed both bandpass and

lowpass filtered nano-SPEAR recordings. "Spot 1" and "Spot 2" refer to two different fluorescent puncta on muscle cells near the nano-SPEAR. In all cases, we found that the distribution of peak correlogram values in shuffled and unshuffled data showed no significant difference, indicating that our nano-SPEAR recordings do not correlate with worm movement ($p > 0.05$, unpaired, two-sided Welch's t-test comparing the means of each distribution). The data represent recordings from a single animal, but similar results were found in $n = 2$ animals.

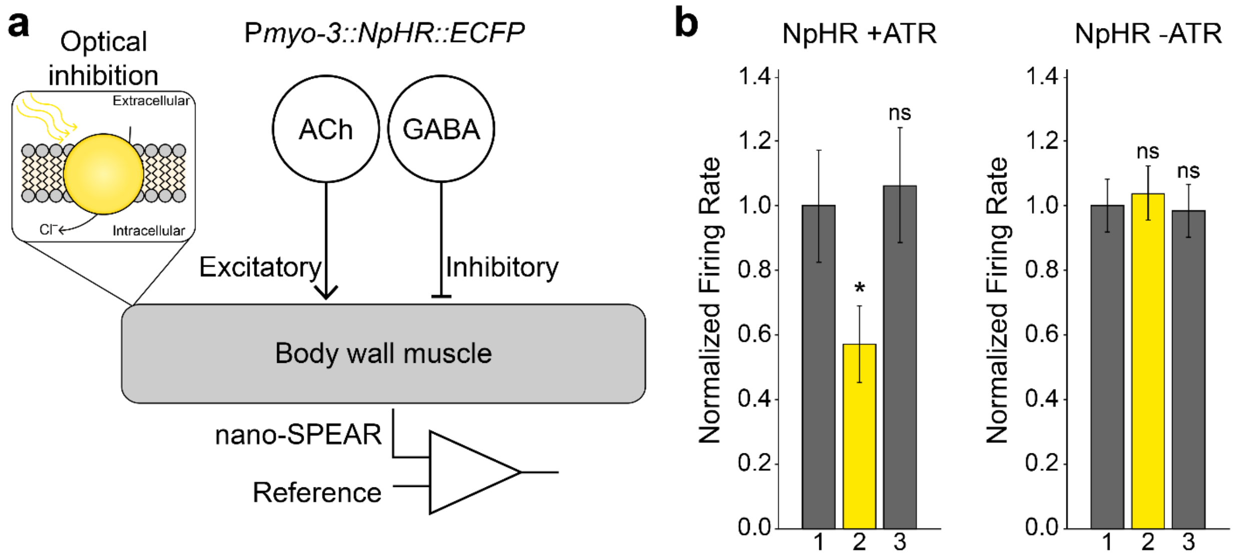

Supplementary Fig. 4 | Nano-SPEARs record a drop in firing rate when body-wall muscles are optically inhibited. a, An illustration of the body-wall neuromuscular junction displaying NpHR expressed in the muscle cells (*myo-3* promoter). **b,** Mean firing rates for three intervals in which there is a 5 s period of no illumination (1), 5 s period of yellow-light illumination (2) and a final 5 s period no illumination (3) (see Methods). Test worms raised with ATR (NpHR +ATR) show a significant drop in firing rate during illumination while the control worms raised without ATR (NpHR -ATR) show no significant change in activity under yellow-light illumination (n = 15 for both sets of animals, each animal was tested 8 times; $*p < 0.05$, ns = not significant, one-way ANOVA with a *post hoc* Bonferroni test compared to Interval 1, error bars are the standard error). Notably, only muscle cells are inhibited in this experiment while presynaptic electrophysiology remains active.

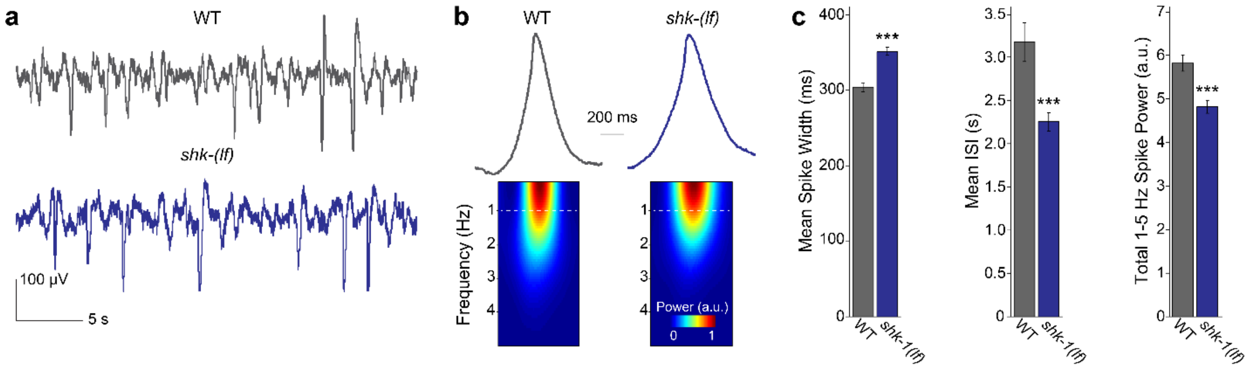

Supplementary Fig. 5 | nano-SPEARs measure longer waveforms in *shk-1(lf)*. a, Representative traces from WT and *shk-1(lf)* animals. **b,** Mean WT and *shk-1(lf)* waveforms and corresponding spectrograms. **c,** As expected, nano-SPEARs detected a longer *shk-1(lf)* waveform. Unexpectedly, the mean ISI for *shk-1(lf)* decreased, indicating that the narrow trap channel may affect AP generation. It should also be noted that there are conflicting reports regarding the effect of the *shk-1(lf)* mutation on muscle cell ISI. Patch-clamping experiments have reported both longer and negligibly different ISIs when comparing *shk-1(lf)* to WT[1,2]. Finally, the change in spike kinetics translate into differences in power in the 1-5 Hz domain in the waveform spectrograms (**p < 0.01, ***p < 0.001, unpaired Welch's t-test).[13,39]. Finally, the change in spike kinetics translate into differences in power in the 1-5 Hz domain in the waveform spectrograms (**p < 0.01, ***p < 0.001, unpaired, two-sided Welch's t-test, error bars are the standard error).

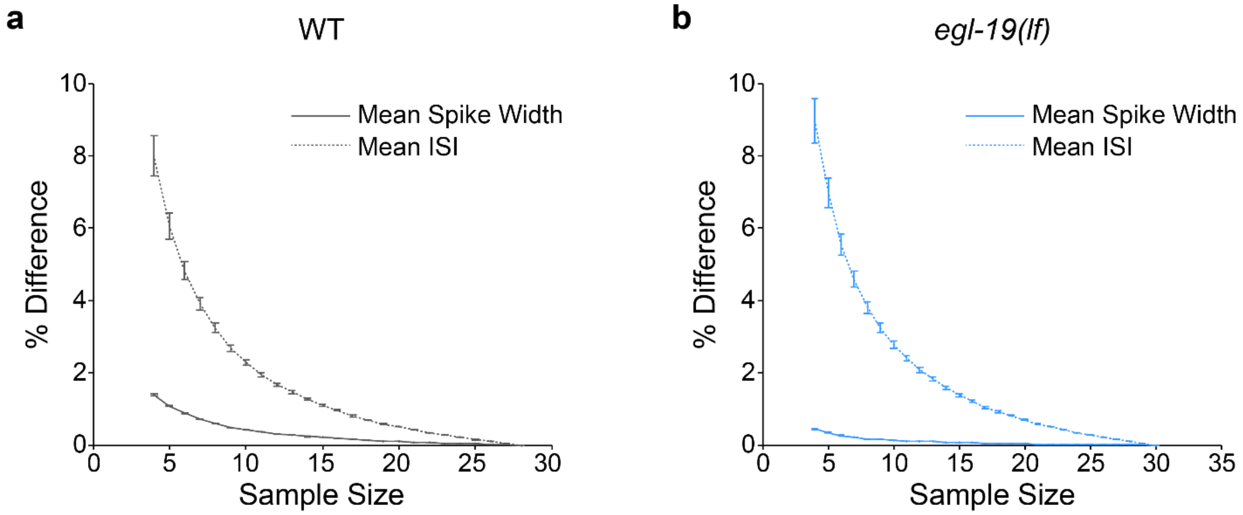

Supplementary Fig. 6 | Quantification of the error involved when sampling a small number of animals. For both plots, the y-axis represents the percent difference from the population average. **a,** Randomly selected subsets from a total of 28 recordings from WT worms show that on average subsets as small as 6 worms closely match the population average (<6% error for spike width and <2% error for ISI). **b,** Similarly, subsets of 30 *egl-19(lf)* worms also show average spike width and ISI values that closely match the population average. These results indicate the sample sizes used in this manuscript (n > 6) are sufficient to phenotype these mutants, which show large (> 20%) differences in these electrophysiological metrics when compared to WT. Error bars are the standard error.

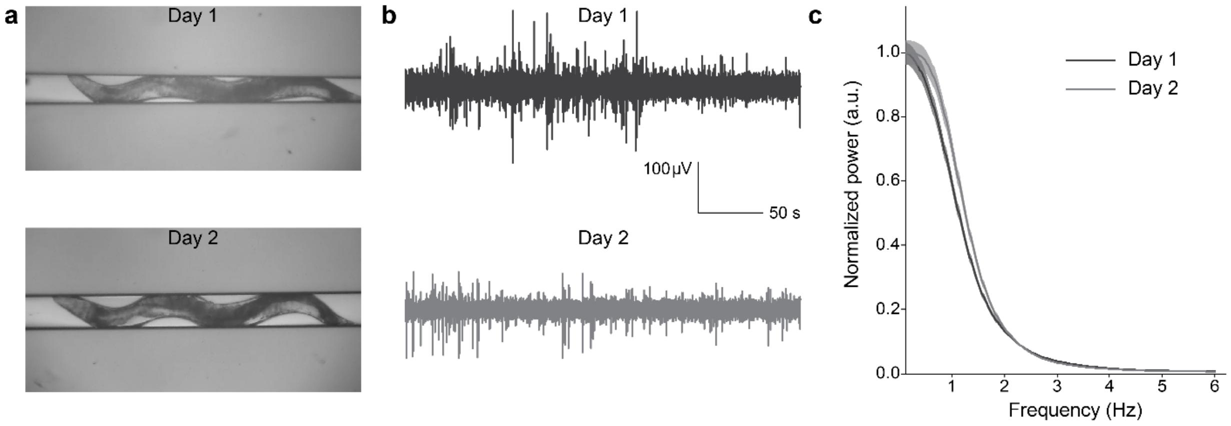

Supplementary Fig. 7 | Recordings on consecutive days from the same worm. a, Optical micrographs shows the same WT worm crawling on day 1 (top) and day 2 (bottom) in a portion of the microchip outside the narrow worm trap. **b,** Bandpass filtered nano-SPEAR recordings from the same animal on day 1 (top) and day 2 (bottom). **c,** We find similar power spectra for the day-1 and day-2 recordings.

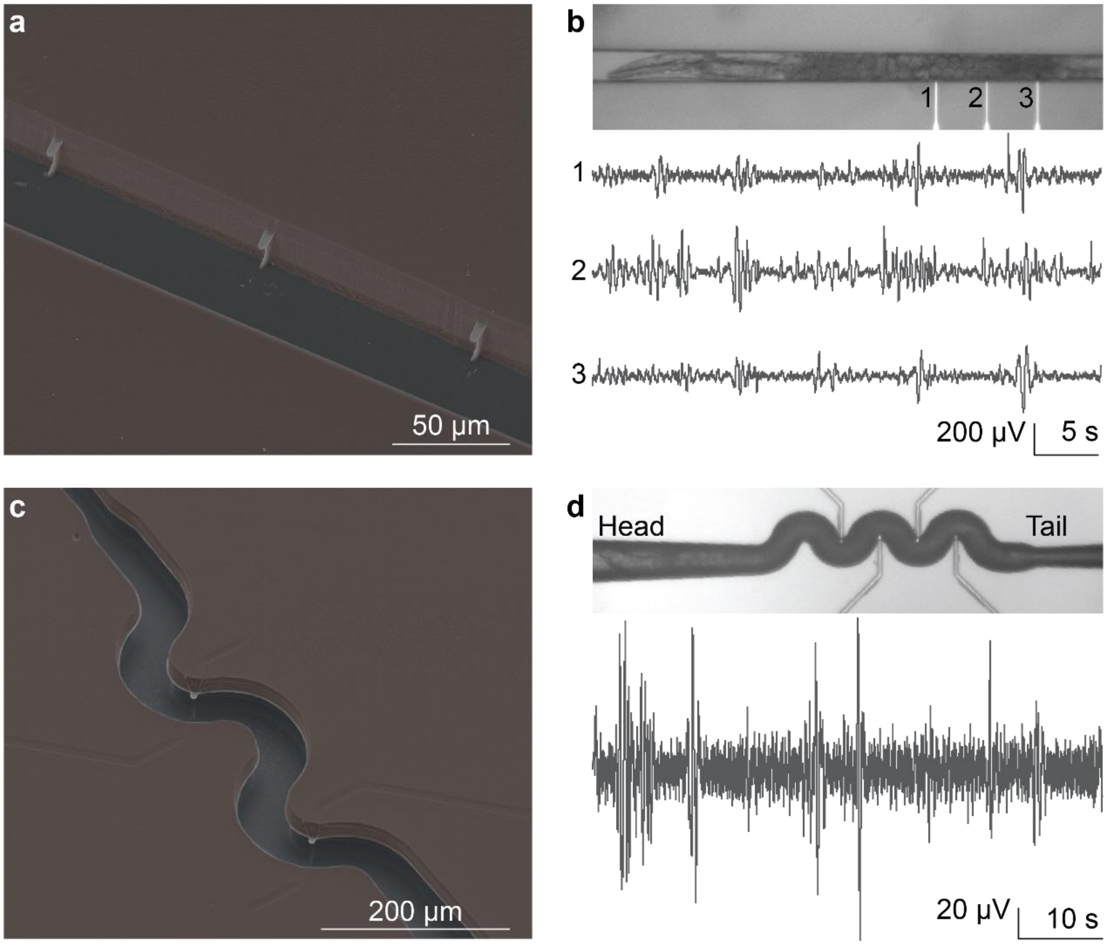

Supplementary Fig. 8 | Diverse trap and electrode configurations for *C. elegans* recording chambers. a, Electron micrograph showing three nano-SPEARs in the same recording chamber on a glass substrate. **b,** (Top) Optical micrograph of a WT animal immobilized against all three electrodes. (Bottom) Simultaneous recordings from the three nano-SPEARs in contact with a single worm. **c,** Electron micrograph of nano-SPEARs in a curved worm trap. **d,** (Top) WT *C. elegans* trapped in a curved chamber. (Bottom) Characteristic nano-SPEAR recording from the curved chamber in (**d**). This recording geometry may increase muscle activity through proprioceptive activation of muscle cells[3].

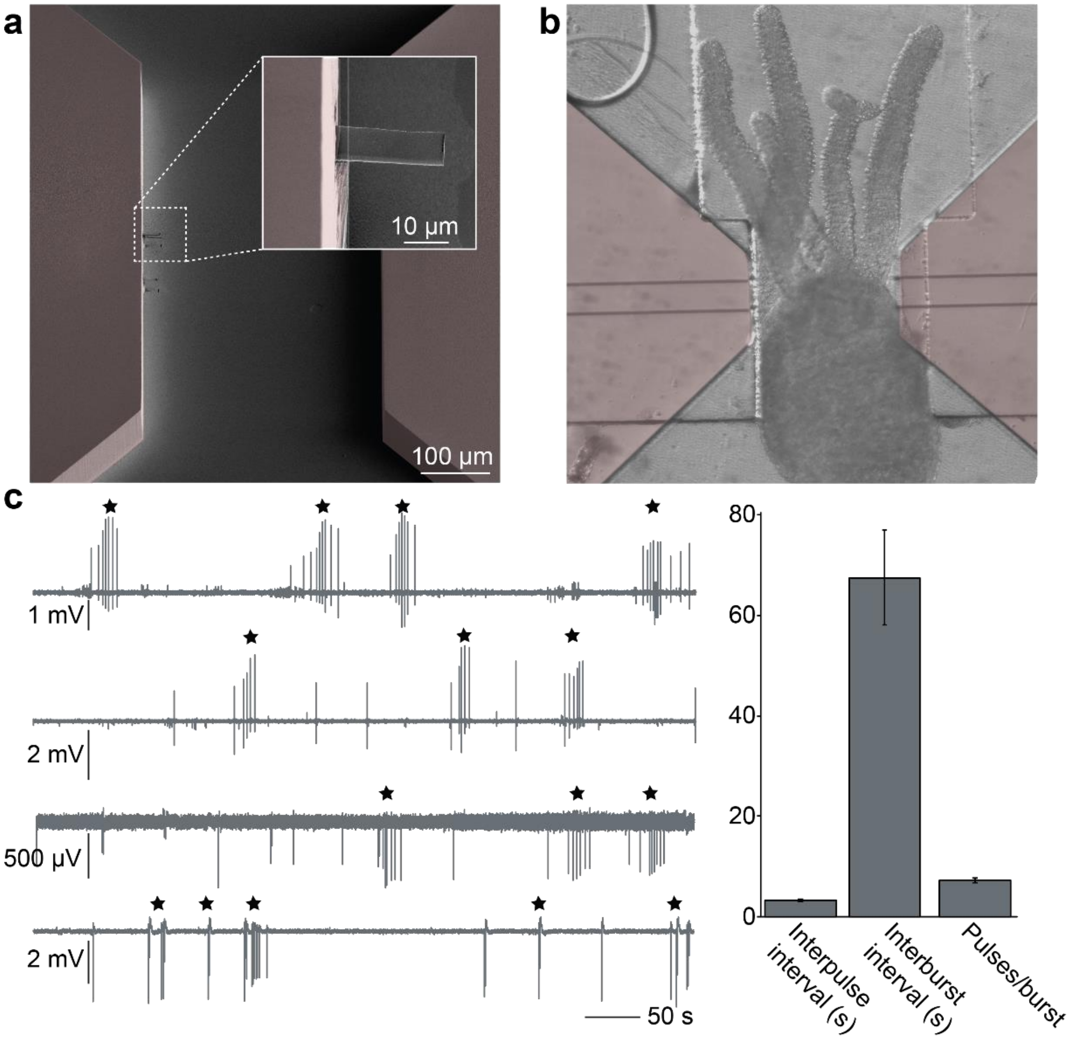

Supplementary Fig. 9 | nano-SPEARs record from *Hydra*. a, Electron micrograph of the recording chamber specifically designed to accommodate *Hydra*. **b,** Micrograph of a *Hydra* immobilized in the recording chamber. **c,** (left) Representative traces from four *Hydra* show distinct, high-amplitude pulses. Stars denote contraction bursts, which are previously reported in *Hydra* recordings[4]. (right) We quantified the mean interpulse interval (the time between contraction burst pulses), the mean interburst interval (the time between contraction bursts), and the average number of pulses per contraction bursts (n = 4, error bars are the standard error).

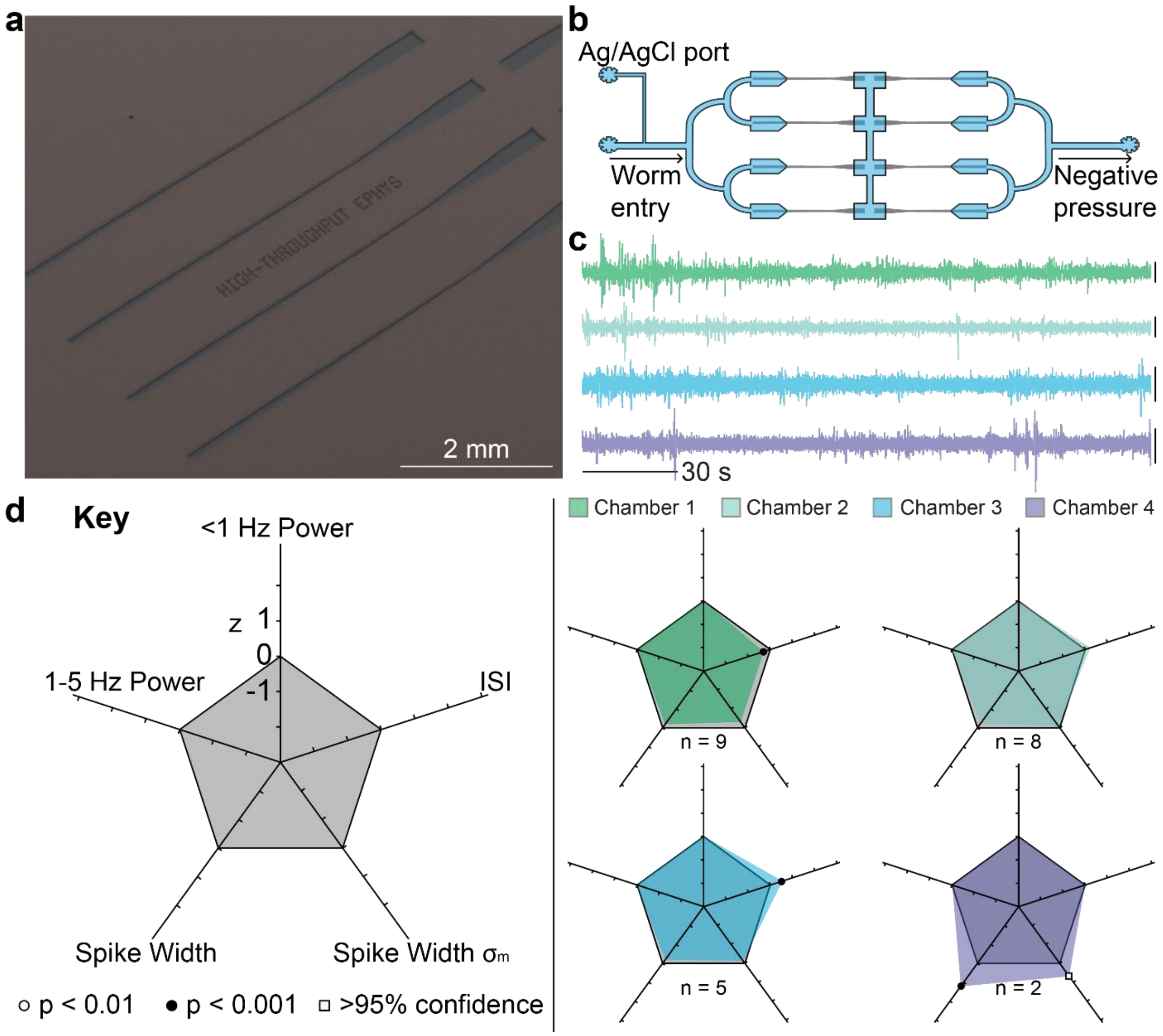

Supplementary Figure 10 | nano-SPEARs for scalable electrophysiology. a, Electron micrograph of an array of *C. elegans* recording chambers. **b,** Schematic showing that the microfluidic interface (blue) can be tailored to interface with multiple recording chambers (gray). Typically only the left or right array of chambers is used for parallel recordings. Arrows indicate the direction of fluid flow used to immobilize animals in the left set of chambers. The flow can be reversed to allow animals to fill the array of chambers on the right. **c,** Representative traces recorded separately from the four chambers used for recordings. Traces are bandpass filtered with cutoff frequencies of 1 and 100 Hz. All vertical scale bars are 100 μV. **d,** We quantified metrics from 24 animals recorded over a period of 1.5 hr. Phenotypic maps (see Methods) compare the five electrophysiology metrics between chambers to display the chamber-to-chamber variability. Each axis is in units of the z-score with respect to the cumulative data of the three other chambers (significance calculated via a Kruskal-Wallis with a *post-hoc* Dunn-Sidak test for power metrics, an unpaired, two-sided Welch's t-test for Spike Width and ISI, and the 95% confidence interval for Spike Width σ_m). We note that slight differences in the recording chambers can lead to small differences in the recorded spike width or ISI, thus for phenotyping experiments we only compare animals recorded in the same chamber. Improvements in manufacturing will likely reduce the chamber-to-chamber variability.

Supplementary Video Captions

Movie 1 | Worms regain normal locomotion soon after immobilization. An animal that has previously been immobilized against a nano-SPEAR for 5 min (left) shows no noticeable change in locomotion from a control animal (right) that has not been immobilized.

Movie 2 | Simultaneous nano-SPEAR recording and bright-field imaging. Simultaneous bright-field imaging of immobilized worms while performing nano-SPEAR recordings show no obvious correlation between worm movement and electrical activity. Dots denote features of the worm that were tracked for quantification (Fig. 2, Supplementary Fig. 3). Sound indicates when spikes are detected. Playback speed is 2X.

Movie 3 | Simultaneous nano-SPEAR recording and fluorescent imaging. Simultaneous imaging of muscle-cell GFP (expressed in nuclei and mitochondria) and nano-SPEAR recordings show no significant correlation between muscle-cell movement and electrical recordings. Sound indicates when spikes are detected. Playback speed is 2X.

References

1. Gao, S. & Zhen, M. Action potentials drive body wall muscle contractions in Caenorhabditis elegans. *Proc. Natl. Acad. Sci. U. S. A.* **108,** 2557–2562 (2011).
2. Liu, P. *et al.* Genetic dissection of ion currents underlying all-or-none action potentials in C. elegans body-wall muscle cells. *J. Physiol.* **589,** 101–117 (2011).
3. Wen, Q. *et al.* Proprioceptive Coupling within Motor Neurons Drives C. elegans Forward Locomotion. *Neuron* **76,** 750–761 (2012).
4. Passano, L. M. & McCullough, C. B. The Light Response and the Rhythmic Potentials of Hydra. *Proc. Natl. Acad. Sci. U. S. A.* **48,** 1376–1382 (1962).
